# Control of Lymphorrhea and Treatment of Warty Excrescences in Elephantiasis

**DOI:** 10.1155/2012/834798

**Published:** 2012-11-18

**Authors:** José Maria Pereira de Godoy, Patricia Amador Franco Brigídio, Edivandra Buzato, Maria de Fátima Guerreiro Godoy

**Affiliations:** ^1^Cardiology and Cardiovascular Surgery Department, Faculty of Medicine School of São José do Rio Preto (FAMERP), Avenida Brigadeiro Faria Lima, 5416 Vila São Pedro, 15090-000 São José do Rio Preto, SP, Brazil; ^2^Vascular Laser Center, Clínica Godoy, 1306 Avenida Constituição, 15025-120 São Jose do Rio Preto, SP, Brazil

## Abstract

The aim of this study is to report the control of lymphorrhea in the intensive treatment of elephantiasis, using an Unna boot. The case of a 29-year-old female patient is reported. This young patient evolved with the more serious form of lymphedema, elephantiasis, after surgical treatment of an abdominal neoplasm and radiotherapy. Warty excrescences were present on both legs and genitalia where lymphorrhea was constant. The patient arrived at the Godoy's Clinic for treatment. She was weighed and perimetric evaluations were made at the start of treatment and thereafter every day during an intensive outpatient treatment of eight hours daily for three weeks. Treatment included manual lymph drainage, mechanical lymph drainage using the RA Godoy device, and the continuous use of compression stockings with adjustments made every three hours. An Unna boot was employed as compression at sites of dermal lesions (warty excrescences) with overlapping use of individualized compression stockings that were individually adapted. The Unna boot was renewed every two days during the first week and every 3 days during the second and third weeks. By the end of this course of treatment, most of the warty excrescences had reduced in size or even disappeared and the lymphorrhea was controlled.

## 1. Introduction

Lymphedema usually affects poor populations; there is no cure and little prospect of therapies being developed by the private health sector. This situation is aggravated in less developed countries where the lack of government resources and specialized health care professionals has led to the marginalization of this disease [1].

An association of therapies, which generally includes manual lymph drainage, compression therapy, exercises, and hygienic care, is recommended for the treatment of lymphedema [1, 2]. More recently other options, such as mechanical lymph drainage employing devices that use either active or passive muscle movements, pressure therapy, daily life activities, and hygienic, nutritional and psychological care, have been added to this arsenal [1, 3, 4].

Intensive treatment of lymphedema, which offers the possibility of the rapid control of swelling, has been reported in the literature [5]. However major problems of patients with elephantiasis are dermal lesions and lymphorrhea that make hygiene and the use of compression, which are essential for treatment, more difficult.

The aim of this study is to report on the use of an Unna boot that allowed the use of an associated compression mechanism with a resulting faster reduction in leg volume, thereby offering a new perspective in the treatment of wartyexcrescences and lymphorrhea in this most severe form of lymphedema.

## 2. Case Report

We report the case of a 29-year-old female patient with lymphedema of the lower limbs. The patient arrived at the Clinica Godoy in Sao Jose do Rio Preto for treatment in January 2011. The patient reported that the lymphedema started at the age of 12 or 13 years old when she was submitted to a surgery to remove a tumor in the abdomen, which started with pain and the diagnosis of appendicitis. During surgery a lymphoma was identified. After surgery, the patient was submitted to chemotherapy and radiotherapy sessions; she does not remember how many sessions due to her age at that time; her mother, who accompanied her, has passed away. The patient reported that the swelling began in the thigh region and spread to the feet; initially the edema got better with rest but eventually this improvement was no longer noted. Her vascular physician at the time prescribed lymph drainage, pressure therapy, and elastic compression hosiery. Even with treatment she noted that the edema increased and fibrosis developed in the abdominal region and eventually she abandoned treatment. With time the edema worsened further and wartyexcrescences began to develop on both legs and the genitalia with constant discharge of lymph. 

The patient was weighed and perimetric evaluations were made at the start of treatment and it was noted that the patient had difficulties to move the legs. Treatment consisted of an intensive (8 hours per day, 5 days per week) program with mechanical lymph drainage using the RAGodoy apparatus, Godoy and Godoy manual lymph drainage, and compression therapy.

The RAGodoy is an electromechanical device that performs passive movements of the ankle joint (dorsiflexion and plantar flexion) adapted to the treatment of lymphedema. This device promotes the deep lymphatic drainage and the Godoy and Godoy lymphatic drainage technique performs manual compression followed by sliding over the lymphatic collectors to the corresponding lymph nodes.

The Unna boots (Unnaflex) is an elastic bandage composed of zinc dioxide (which does not become stiff), glycerin, starches, castor oil, and white petrolatum. It adapts to the contour of the leg stretching softly and remaining flexible and is applied in the same way that one bandage, into spirals movements.

**Figure 1:**
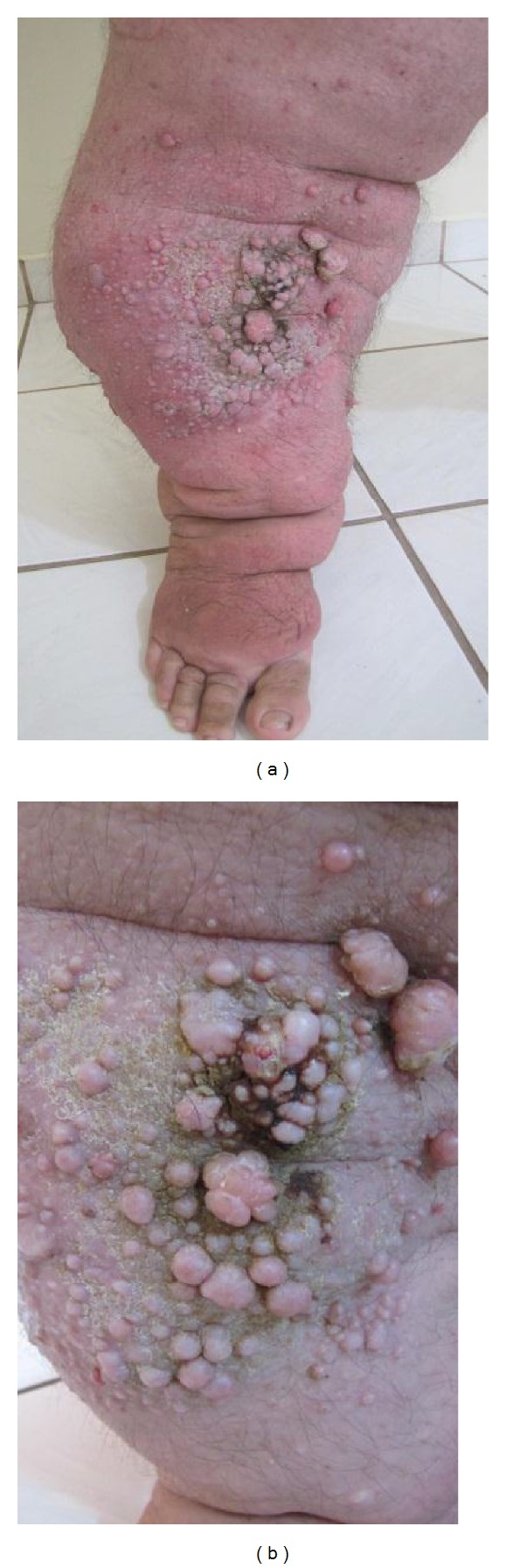
(a), (b): Dermal lesions and lymphorrhea (before treatment).

**Figure 2:**
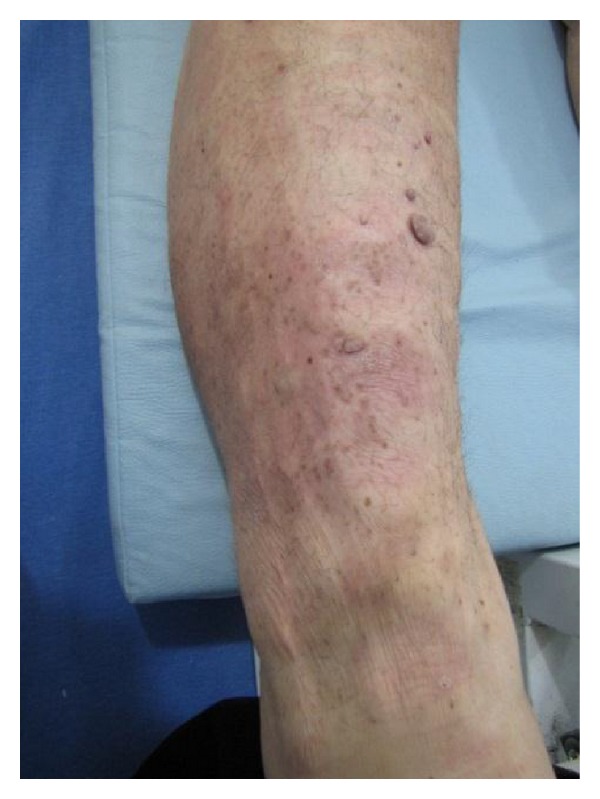
After treatment.

Unna boots were employed on both legs to protect and for compression at the sites of dermal lesions (wartyexcrescences) with overlapping using individualized low-stretch compression stockings adapted to take into account the deformities made from a cotton-polyester fabric. Daily assessments of body weight and leg perimeter were made. The Unna boot was renewed every two days during the first week and every 3 days during the second and third weeks. The boot was employed for three weeks until most of the wartyexcrescences had reduced in size or even disappeared and the lymphorrhea was controlled (Figures 1 and 2).

Major deformities and the skin of the patient were monitored monthly. The patient was advised continuously about the need of hygienic care, to control the edema, and about the normalization of skin lesions.

## 3. Discussion

This study describes an alternative to treating major deformities where wartyexcrescences and lymphorrhea are aggravating factors. The use of compression without skin protection in these patients is associated with infection and worsening of the condition. Friction between the skin and the compression mechanism, without protection using creams, is associated to excoriation and infection.

Recently an Unna boot has been employed in these cases as it protects against infections and allows a reduction in the volume of lymphedema with most wartyexcrescences disappearing during treatment. Even large lesions generally disappear spontaneously; when they do not, they can be eliminated by cauterization. In this case the approach was possible because the lesions were isolated; however when there are several lesions together, the friction between them may result in infections due to the development of skin injuries.

The compression exerted by Unna boots is nonelastic and so useful in the treatment of lymphedema. The patient was prescribed penicillin-based antibiotics during this period as prophylaxis. This intensive approach allows large volume reductions of the legs; this patient lost about 31 kilos in 10 days of treatment. Thus, this quick reduction in weight helps to avoid injuries related to the wartyexcrescences which initially become flat and gradually disappear.

This clinical approach reduced the necessity of surgical interventions of these excrescences, controlling lymphorrhea and protecting against the use of nonelastic compression mechanisms.

We concluded that Unna boot is an option in the protection of warty excrescences and in control of lymphorrhea in patients with elephantiasis during the treatment of edema.
